# The Diseases of Children

**Published:** 1858-05

**Authors:** 


ART. VI.—*The Diseases of Children.* By FLEETWOOD CHURCHILL, M.D., &c. Second edition. Dublin: Fannin & Co. 8vo. pp. 782. 1858.

THE appearance of a new edition of this work so soon after the publication of the first, affords sufficient evidence of the estimation in which it is held by the profession. Its predecessor received the commendation of most of the medical press, both of Great Britain and this country, in the latter of which it was honored with a reprint by Messrs. Blanchard & Lea. Although the profession of the United States was already in possession of able treatises on diseases of children by Condie, Meigs, and Stewart, yet the work of Dr. Churchill was calculated, by its great erudition and completeness, to fill a void that had long been felt in our medical literature on this subject. Without laying any claims to originality, it brings before the reader an amount of information not comprised in any similar production in the language. The amount of labor consumed upon its production can only be conceived by those who have been similarly occupied, every work of note published within the last twenty-five years in the different languages of Europe, having been laid under contribution for the illustration of its topics. The material thus derived has been used with consummate skill, and the result has been a work creditable alike to the author and his country. The present edition comprises one hundred and thirty pages of new matter, and the discussion of a number of new topics not included in its predecessor. The most important of the new chapters treat of syphilis, phthisis, and paralysis.

